# Absence of genome reduction in diverse, facultative endohyphal bacteria

**DOI:** 10.1099/mgen.0.000101

**Published:** 2017-02-28

**Authors:** David A. Baltrus, Kevin Dougherty, Kayla R. Arendt, Marcel Huntemann, Alicia Clum, Manoj Pillay, Krishnaveni Palaniappan, Neha Varghese, Natalia Mikhailova, Dimitrios Stamatis, T. B. K. Reddy, Chew Yee Ngan, Chris Daum, Nicole Shapiro, Victor Markowitz, Natalia Ivanova, Nikos Kyrpides, Tanja Woyke, A. Elizabeth Arnold

**Affiliations:** ^1^​ School of Plant Sciences, University of Arizona, Tucson, AZ 85721, USA; ^2^​ Joint Genome Institute, Walnut Creek, CA, USA; ^3^​ Department of Ecology and Evolutionary Biology, University of Arizona, Tucson, AZ 85721, USA

**Keywords:** endofungal bacteria, endosymbiont, horizontal transmission, *Luteibacter*, endohyphal bacteria

## Abstract

Fungi interact closely with bacteria, both on the surfaces of the hyphae and within their living tissues (i.e. endohyphal bacteria, EHB). These EHB can be obligate or facultative symbionts and can mediate diverse phenotypic traits in their hosts. Although EHB have been observed in many lineages of fungi, it remains unclear how widespread and general these associations are, and whether there are unifying ecological and genomic features can be found across EHB strains as a whole. We cultured 11 bacterial strains after they emerged from the hyphae of diverse Ascomycota that were isolated as foliar endophytes of cupressaceous trees, and generated nearly complete genome sequences for all. Unlike the genomes of largely obligate EHB, the genomes of these facultative EHB resembled those of closely related strains isolated from environmental sources. Although all analysed genomes encoded structures that could be used to interact with eukaryotic hosts, pathways previously implicated in maintenance and establishment of EHB symbiosis were not universally present across all strains. Independent isolation of two nearly identical pairs of strains from different classes of fungi, coupled with recent experimental evidence, suggests horizontal transfer of EHB across endophytic hosts. Given the potential for EHB to influence fungal phenotypes, these genomes could shed light on the mechanisms of plant growth promotion or stress mitigation by fungal endophytes during the symbiotic phase, as well as degradation of plant material during the saprotrophic phase. As such, these findings contribute to the illumination of a new dimension of functional biodiversity in fungi.

## Abbreviations

DOE, United States Department of Energy; EHB, endohyphal bacteria; IMG, Integrated Microbial Genomes; JGI, Joint Genome Institute; SNP, single nucleotide polymorphism.

## Data Summary

Whole-genome assemblies for all isolates have been deposited in GenBank with the accession numbers listed in Table 1. We have listed the sequencing and assembly summaries on Figshare at https://dx.doi.org/10.6084/m9.figshare.4123320. The accession numbers for all genomes used in this report, KEGG classifications and genome size calculations can be found on Figshare, as above. Protein alignments and output files for phylogenetic comparisons can also be found on Figshare, as above. Raw sequencing reads are accessible on the United States Department of Energy Joint Genome Institute Integrated Microbial Genomes (IMG) system (https://img.jgi.doe.gov/) by querying the IMG ID numbers listed in Table 1.

**Table 1.** Genome characteristics of endohyphal bacteria

	*Burkholderia* 9120	*Curtobacterium* 9128	*Erwinia* 9145	*Luteibacter* 9133	*Luteibacter* 9135	*Luteibacter* 9143	*Luteibacter* 9145	*Massilia* 9096	*Pantoea* 9133	*Pantoea* 9140	*Rhizobium* 9140
Genome size (bp)	8455632	3943501	4254300	4501157	4485268	4625266	4559593	5710276	4673532	4839470	5428940
Gene count	7578	3768	4120	3986	3839	4204	4111	5068	4464	4602	5228
Contigs and scaffolds	2	1	1	1	1	1	1	2	2	3	7
mol% G+C	62.73 %	70.57 %	55.18 %	64.90 %	66.24 %	64.84 %	64.87 %	65.70 %	55.38 %	55.30 %	62.52 %
% DNA coding bases	87.25 %	91.88 %	89.68 %	91.71 %	91.47 %	91.69 %	91.73 %	89.84 %	88.05 %	87.94 %	89.04 %
GenBank accession no.	JQNA00000000.1	LT576451.1	JQNE00000000.1	JUHO00000000.1	JQNB00000000.1	JQNL00000000.1	JQND00000000.1	JQNN00000000.1	FKKH00000000.1	JQNO00000000.1	FJUR00000000.1
JGI IMG ID no.	2582580720	2602042068	2582581251	2585427589	2582580721	2582581258	2582581226	2582581239	2602042078	2582581300	2602042090
Fungal host of isolation	*Monodictys* sp.	*Cladosporium* sp.	*Microdiplodia* sp.	*Botryosphaeria* sp.	*Phyliostricta* sp.	*Pestalotiopsis* sp.	*Microdiplodia* sp.	*Hormonema* sp.	*Botryosphaeria* sp.	*Microdiplodia* sp.	*Microdiplodia* sp.

## Impact Statement

Fungi can harbour symbiotic bacteria inside their hyphae, and the presence of these bacteria has been shown to alter the phenotypes of the fungal hosts. Most known endohyphal bacteria (EHB) are vertically transmitted obligate symbionts characterized by reduced genomes. In comparison to previously described strains, here we report genome sequences for a diverse array of EHB isolated from foliar endophytes of cupressaceous plants. These symbionts appear to be facultative, and show few (if any) signs of genome reduction. Furthermore, a lack nucleotide diversity across two strains isolated from fungi in different classes suggests either horizontal transmission or relatively recent independent acquisitions from a common environmental reservoir. The absence of conserved molecular pathways mediating bacterial–fungal symbioses highlights the differences between these facultative EHB and previously described obligate symbionts. Taken together, data presented here hint at the complexities in the partnerships between microbes and fungi under natural conditions and highlights how phenotypic plasticity in fungi could be mediated by a variety of EHB.

## Introduction

All eukaryotes have evolved in the presence of bacteria, with diverse bacteria adopting an endosymbiotic and intracellular habitat across the eukaryotic tree of life [1, 2]. Much like the diverse Metazoa that host rich bacterial microbiomes, fungi interact closely with bacteria both on the surfaces of hyphae and within their living hyphae (i.e. endofungal or endohyphal bacteria, EHB) [3–7]. These EHB can be either obligate or facultative symbionts and can mediate diverse phenotypic traits in their hosts. For instance, EHB inhabiting some rhizosphere fungi can influence the virulence of some phytopathogens, the capacity of certain mycorrhizal fungi to establish symbiotic associations and could potentially also affect nutrient acquisition [6, 8, 9]. In turn, EHB inhabiting foliar fungal endophytes (fungi that occur in living leaves without causing disease; class 3 endophytes, *sensu* Rodriguez *et al*. [10]) can increase the production of plant growth-promoting hormones [11] and alter the capacity of their hosts to degrade plant tissues [12]. EHB have been observed in many of the major lineages of plant-associated fungi (including diverse Mucoromycotina, Glomeromycota, Basidiomycota and Ascomycota), and we have recently demonstrated that plant-associated Ascomycota potentially harbour a vast array of bacteria, albeit ephemerally in some conditions [13]. Despite these observations, questions remain about the life cycles for the majority of culturable bacteria found within fungal hyphae, and whether unifying ecological and genomic traits are common among all EHB strains. To better understand the genomic characteristics of facultative EHB associated with fungal endophytes, we isolated 11 bacterial strains after they emerged from the hyphae of diverse Ascomycota that were isolated as endophytes of cupressaceous plants, and generated nearly complete genome sequences for all.

Multiple EHB associated with other fungal taxa have already been sequenced and analysed in genome-level studies (e.g. [5, 7, 14]), providing a framework for determining whether previously identified genomic trends hold across all EHB symbionts. At present, the best-understood system for exploring interactions between EHB and fungal hosts focuses on *Burkholderia rhizoxinica* and the plant pathogen *Rhizopus microsporus*. Within this symbiosis, the bacterium produces a toxin required for fungal pathogenicity on rice [6]. Notably, this symbiosis appears to be maintained and established by a chitinase secreted by a type II secretion system [15], unknown products secreted by a type III system [16], and unusual modifications to the lipopolysaccharide of the bacterium [17]. Furthermore, *B. rhizoxinica* can be transmitted vertically through fungal spores, ensuring close association of the fungal host and bacterial symbiont across generations [18]. The biology of Mollicutes-related endobacteria of Glomeromycota is largely unknown [5, 7], whereas ‘*Candidatus* *Glomeribacter* *gigasporarum*’, also in Glomeromycota, directly modifies fungal stress responses through unknown mechanisms [19]. Lastly, the plant pathogen *Ralstonia solanacearum* is able to induce production of and then invade chlamydospores of *Aspergillus* spp., with both processes influenced by the production of secondary metabolites by the bacterium [20].

One of the dominant features found across EHB genomes sequenced to date is a reduction of genome size and coding capacity relative to the genomes of non-EHB outgroup strains. Genome reduction is thought to occur within many obligate symbionts and parasites, because population bottlenecks during vertical transmission increase the fixation of deleterious mutations via genetic drift (reviewed by Martinez-Cano *et al.* [21]). In general, the environment experienced by obligate symbionts is also more constant than that of free-living bacteria, resulting in weaker selection pressures for maintenance of some protein-encoding capacities and various biochemical pathways. These trends, coupled with an overall deletion bias across bacterial genomes, are thought to result in reduced genome sizes for strains that live obligately inside of hosts. Alternatively, genome size reduction could be the product of selection pressures for ‘streamlined’ genomes in bacteria that have large population sizes and experience environments that are limiting for critical nutrients or where ‘leaky’ pathways are metabolically costly [22, 23]. The genome size of *B. rhizoxinica* is quite reduced compared to that of free-living congeners [14], as is that of a related symbiont of the fungus *Mortierella elongata* [24]. Similarly, genomes of Mollicutes-related endobacteria (which are also obligate symbionts of fungi) are relatively small compared to those of free-living bacteria [5, 7]. However, genomes of Mollicutes are generally small, such that size reductions may have occurred before evolution of the endofungal lifestyle [25].

Previous efforts linking bacterial genome size to ecological variables across systems have suggested that larger genomes could better buffer bacterial populations against changing environments, so that one might expect facultative symbionts to have larger genomes than obligate symbionts that only experience one host [26]. Given clear patterns of genome reduction demonstrated across obligate fungal symbionts, we posited that analysis of genome sizes within a phylogenetic context could provide important ecological clues to better understand the life cycles of facultative EHB. In contrast to the genomes of previously studied, largely obligate EHB, here we found that the sizes of genomes from facultative EHB resembled those of closely related strains isolated from environmental sources. Furthermore, while these EHB strains all possessed structures that were capable of interacting with eukaryotic hosts, we did not find evidence for a conserved pathway that mediated EHB–fungal interactions across all strains. We consider these genome data to be informative regarding little-known aspects of the transmission and population dynamics of EHB. Because EHB can influence plant growth promotion by fungal endophytes during the symbiotic phase [11], as well as degradation of plant material during the saprotrophic phase [12], these genomes could enable engineering of symbiotic associations to enhance the growth and processing of plant material.

## Methods

### Isolation of bacterial strains and genomic DNA

To trigger emergence of bacterial strains from their fungal hosts, mycelia were grown from plugs on 2 % malt extract agar at 36 °C [3, 4, 11]. After 72 h, bacteria generally emerged from apparently axenic mycelium. The endohyphal status of all bacteria was confirmed prior to emergence following the methods of Hoffman and Arnold [4] and Arendt *et al.* [3] through PCR and light microscopy. In all but one case, emergent bacteria were streaked to single colonies on lysogeny broth (LB) media without antibiotic supplements. *Rhizobium* sp. 9140 was streaked instead to yeast extract mannitol (YEM) medium without antibiotic supplements. Individual colonies were grown in liquid LB media (or YEM broth for *Rhizobium* sp. 9140) and frozen in 40 % (v/v) glycerol, except for *Massilia* sp. 9096, which was frozen in 10 % DMSO. Bacterial strains and genomic DNA were verified through PCR and Sanger sequencing of the 16S rDNA locus using primers 27F and 1492R (see the work of Hoffmanand Arnold [4]). Before isolating genomic DNA, bacterial strains were streaked from frozen stocks, at which point a single colony was inoculated into 5 ml LB media (or YEM broth for *Rhizobium* sp. 9140) and grown at 27 °C overnight. Genomic DNA from this 5 ml culture was isolated using a Wizard genomic DNA isolation kit, following the manufacturer’s instructions (Promega).

### Genome sequencing and assembly

Draft and complete genomes were generated at the United States Department of Energy Joint Genome Institute (DOE JGI) using the Pacific Biosciences (PacBio) sequencing technology [27]. A PacBio SMRTbell library was constructed and sequenced on the PacBio RS platform. Characteristics of each sequencing run and assembly can be found in Table 1, and sequencing and assembly summaries for each genome can be found on Figshare (https://dx.doi.org/10.6084/m9.figshare.4123320). All general aspects of library construction and sequencing performed at the JGI can be found by querying the JGI ID for each strain (Table 1) at www.jgi.doe.gov to pull up each specific project page. Raw reads were assembled using hgap (version 2.2.0.p1) [28].

### Genome annotation

Genomes were annotated using the JGI microbial annotation pipeline [29], followed by a round of manual curation using GenePRIMP [30] for finished genomes and draft genomes in fewer than 10 scaffolds. Predicted coding sequences were translated and used to search the National Center for Biotechnology Information non-redundant database, as well as the UniProt, TIGRFam, Pfam, KEGG, COG and InterPro databases. The tRNAScanSE tool [31] was used to find tRNA genes, whereas rRNA genes were found by searches against models of rRNA genes built from SILVA [32]. Other non-coding RNAs, such as the RNA components of the protein secretion complex and the RNase P, were identified by searching the genome for the corresponding Rfam profiles using infernal [33]. Additional gene prediction analysis and manual functional annotation was performed within the Integrated Microbial Genomes (IMG) platform (http://img.jgi.doe.gov) developed by the JGI [34]. All additional genomic analyses, including those regarding pathway presence and absence, were carried out using the IMG platform.

### Phylogenetic and comparative genomic analyses

**Fig. 1.**
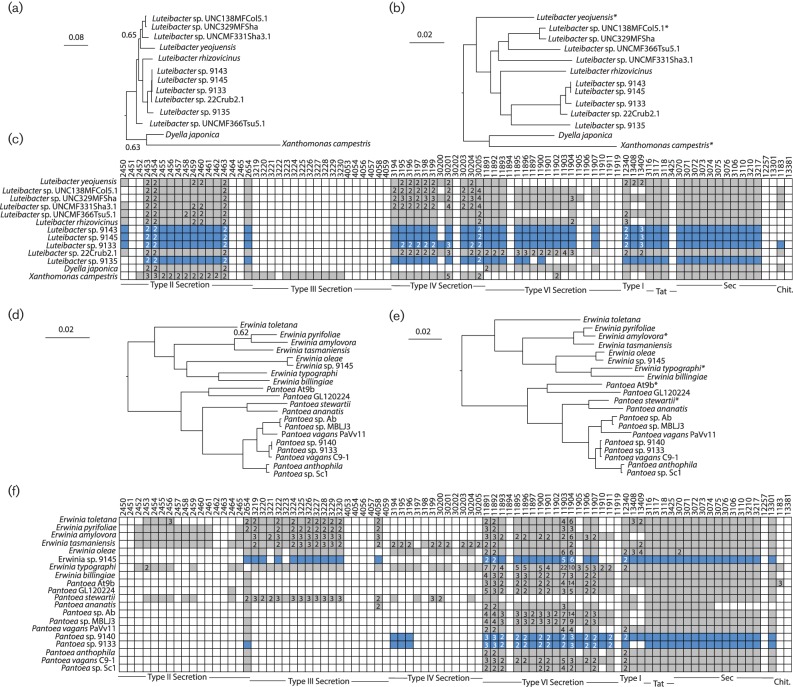
Phylogenetic analysis and comparison of interdomain interaction systems for *Erwinia, Pantoea* and *Luteibacter*. (a, d) Bayesian phylogenies for focal EHB and non-EHB strains for *Leutibacter* (a) or *Erwinia*/*Pantoea* (d) were inferred from concatenated sequences of RpoD and GyrB. Unless noted, posterior probabilities at all nodes are >0.95. (b, e) Maximum-likelihood phylogenies for EHB and non-EHB strains were inferred from whole-genome sequences using RealPhy for *Leutibacter* (b) or *Erwinia*/*Pantoea* (e). (c, f) KEGG pathway searches were implemented in IMG to identify bacterial pathways known to be involved in signalling between bacteria and eukaryotes for *Leutibacter* (c) or *Erwinia*/*Pantoea* (f). Genomes queried for each clade are listed across the *y*-axis. Boxes along the *x*-axis indicate KEGG pathway identifiers (top) for constituent genes for each bacteria secretion system with grouping by system (bottom). Coloured/filled boxes indicate that at least one gene within the genome is present and classified according to that specific KEGG identifier. Numbers inside the coloured/filled boxes denote that more than one gene within that genome is classified according to that KEGG identifier. The boxes for EHB bacteria described in this report are coloured blue. *Indicates that these genomes were used as references for building phylogenies using RealPhy.

**Fig. 2.**
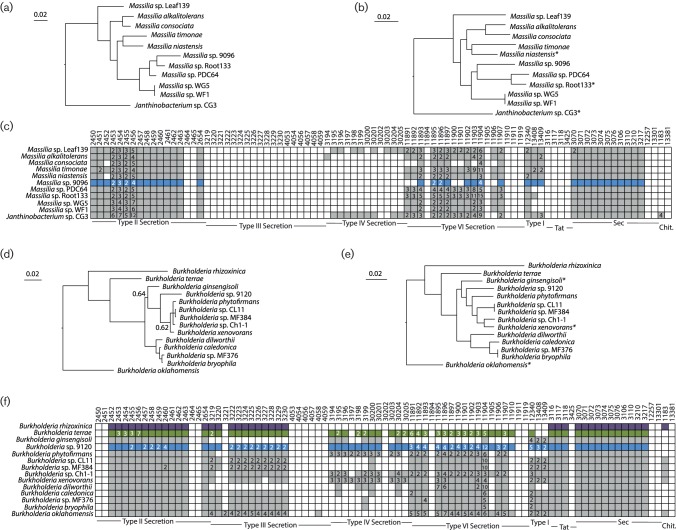
Phylogenetic analysis and comparison of interdomain interaction systems for *Massilia* and *Burkholderia*. (a, d) Bayesian phylogenies for focal EHB and non-EHB strains for *Massilia* (a) or *Burkholderia* (d) were built from concatenated sequences of RpoD and GyrB. Unless noted, posterior probabilities at all nodes are >0.95. (b, e) Maximum-likelihood phylogenies for EHB and non-EHB strains were inferred from whole-genome sequences using RealPhy for *Massilia* (b) or *Burkholderia* (e). (c, f) KEGG pathway searches were implemented in IMG to identify bacterial pathways known to be involved in signalling between bacteria and eukaryotes for *Massilia* (c) or *Burkholderia* (f). Genomes queried for each clade are listed across the *y*-axis. Boxes along the *x*-axis indicate KEGG pathway identifiers (top) for constituent genes for each bacteria secretion system with grouping by system (bottom). Coloured/filled boxes indicate that at least one gene within the genome is present and classified according to that specific KEGG identifier. Numbers inside the coloured/filled boxes denote that more than one gene within that genome is classified according to that KEGG identifier. Boxes for EHB bacteria described in this report are coloured blue. Those for a previously described EHB (*B. rhizoxinica* [14]) or bacteria demonstrated to interact with fungi (*B. terrae* [54]) are coloured purple and green, respectively. * Indicates that these genomes were used as references for building phylogenies using RealPhy.

**Fig. 3.**
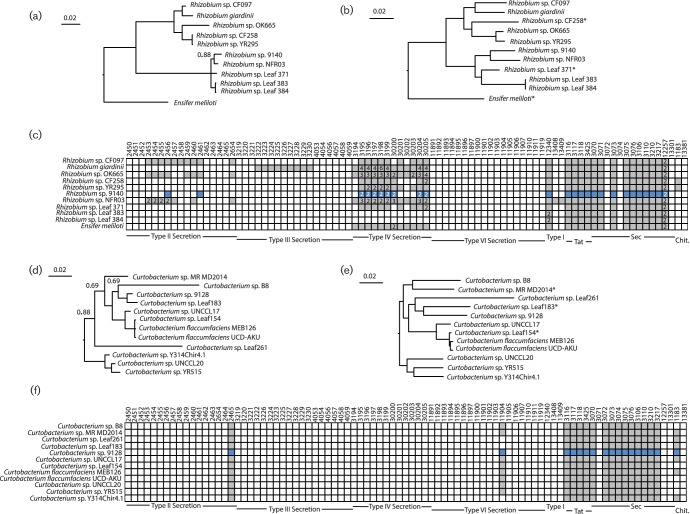
Phylogenetic analysis and comparison of interdomain interaction systems for *Rhizobium* and *Curtobacterium*. (a, d) Bayesian phylogenies for focal EHB and non-EHB strains were built from concatenated sequences of RpoD and GyrB (*Rhizobium*, a) or just GyrB (*Curtobacterium*, d). Unless noted, posterior probabilities at all nodes are >0.95 (b, e) maximum-likelihood phylogenies for EHB and non-EHB strains were inferred from whole-genome sequences using RealPhy for *Rhizobium* (b) or *Curtobacterium* (e). (c, f) KEGG pathway searches were implemented in IMG to identify bacterial pathways known to be involved in signalling between bacteria and eukaryotes for *Rhizobium* (c) or *Curtobacterium* (f). Genomes queried for each clade are listed across the *y*-axis. Boxes along the *x*-axis indicate KEGG pathway identifiers (top) for constituent genes for each bacteria secretion system with grouping by system (bottom). Coloured/filled boxes indicate that at least one gene within the genome is present and classified according to that specific KEGG identifier. Numbers inside the coloured/filled boxes denote that more than one gene within that genome is classified according to that KEGG identifier. Boxes for EHB bacteria described in this report are coloured blue. *Indicates that these genomes were used as references for building phylogenies using RealPhy.

Whole-genome files for all strains listed in Figs 1, 2 and 3 are publicly available through GenBank (see Table 1), as well as through the JGI IMG (see strain information in the Excel spreadsheet at Figshare https://dx.doi.org/10.6084/m9.figshare.4123320), with sequencing and assembly reports for EHB also listed on Figshare (https://dx.doi.org/10.6084/m9.figshare.4123320). Genomes were chosen for comparative analysis by querying GyrB and RpoD sequences using blastp against all sequenced bacterial genomes at the JGI IMG database. Genomes from the top 15–20 hits for each strain lineage were then selected for downstream phylogenetic comparisons. Bayesian phylogenies were built (see below), and strains were culled from this genome set if they were found to clearly be members of separately diverged phylogenetic lineages from EHB strains. In some cases, clear outgroup strains were also included to root analyses.

Bayesian phylogenies were created using protein sequences from conserved genes for each clade. In almost every case, GyrB and RpoD sequences were independently aligned using clustalx [35] and then concatenated, the exception being *Curtobacterium* sp. 9128 (only GyrB was used). MrBayes was used for Bayesian phylogenetic analysis on these sequences [36], using flat priors and a burn-in period of 125 000 generations. In each case, convergence of the run occurred before 500 000 total generations. Alignments and output files from MrBayes can be found on Figshare (at https://dx.doi.org/10.6084/m9.figshare.4123320).

Phylogenies for whole genomes were inferred using the RealPhy online server [37]. Accession identification for non-EHB genomes can be found in a spreadsheet available at Figshare (https://dx.doi.org/10.6084/m9.figshare.4123320). Briefly, for each phylogeny shown in Figs 1, 2 and 3, GenBank files were uploaded to the server and maximum-likelihood phylogenies were built from whole-genome alignments to a single reference genome. Reference phylogenies were built to all strains denoted with an asterisk and then merged to produce the final phylogeny, with at least three reference genomes picked for each analysis. Reference genomes were picked to represent diversity across non-EHB bacteria.

Geneious version 6.0.5 [38] was used to compare whole-genome alignments for *Erwinia* sp. 9140 and *Erwinia* sp. 9145, and *Luteibacter* sp. 9143 and *Luteibacter* sp. 9145. Briefly, sequences from these genomes were aligned using the Mauve option in Geneious with default parameters. Single nucleotide polymorphisms (SNPs) and INDELs were displayed as disagreements between these alignments, were inspected visually for proper alignment, and were counted by eye.

## Results and Discussion

### Convergent evolution of closely related EHB

Whole-genome sequences can provide a broader picture of evolutionary relationships among bacterial strains than phylogenies built from single loci [39]. We inferred phylogenies for our focal strains and related bacteria based on a subset of conserved loci, as well as whole-genome data. Both approaches yielded similar results in terms of phylogenetic placement of EHB and non-EHB strains. Our evaluation of multiple EHB strains from diverse Ascomycota has provided insights into phylogenetic signals associated with the EHB lifestyle, an opportunity to explore shared genomic architecture relevant to the EHB lifestyle, and the opportunity to evaluate whether these EHB have genomic traits consistent with convergent evolution.

For most of our focal EHB, the data suggest that the facultative endohyphal lifestyle has evolved multiple times amongst closely related bacteria. For instance, we found phylogenetically distinct strains of *Erwinia* and *Pantoea* within different classes of fungal hosts (Fig. 1, Table 1). Furthermore, our data demonstrated that the *Burkholderia* sp. 9120 strain whose genome is reported here is phylogenetically distinct from the previously characterized EHB *B. rhizoxinica* (Fig. 2) and *Burkholderia terrae*, which forms a close relationship with fungi from soil [40, 41]. Our *Rhizobium*, *Curtobacterium* and *Massilia* isolates are, to the best of our knowledge, the first from these clades to be recorded as EHB, although all are closely related to strains that associate with plants and which are commonly found in environmental samples (Figs 2 and 3). It remains a possibility that many different environmental bacteria can associate with fungi as endophytes and therefore transiently be categorized as EHB. For example, it is possible that all *Pantoea* and *Erwinia* strains could be found as EHB if sampling of the total population of EHB was possible. Under this scenario, phylogenetic signals of convergence across sequenced strains in this report could simply represent sampling bias. However, we note that our previous categorization based solely on 16S rDNA across a wider variety of fungal hosts also showed clustering of EHB strains into particular clades rather than the presence of diverse sequences from throughout the *Pantoea*/*Erwinia* phylogeny [4, 13].

Compared to the genomes of other facultative EHB, *Luteibacter* strains displayed an interesting phylogenetic pattern that suggests some level of host specificity (Fig. 1). There were two distinct clades within the *Luteibacter* phylogeny: one that was mainly composed of rhizosphere isolates and one that was composed mainly of EHB. Interestingly, this pattern held even when geographical provenance was incorporated, as almost all EHB strains were isolated at Duke Forest (Durham, NC, USA), and many rhizosphere strains were isolated at nearby Mason Farm (Chapel Hill, NC, USA). Of the analysed strains, the only geographical outlier for this pattern was EHB strain 9135, which was isolated in Arizona (USA) but clustered with other EHB to the exclusion of most rhizosphere strains. Likewise, the only (presumably) non-EHB *Luteibacter* strain nested within the EHB *Luteibacter* clade was the rhizosphere strain 22Crub2.1. It is unclear whether this relatively clear phylogenetic clustering of *Luteibacter* EHB represents specialization to the EHB lifestyle or whether it is due to overall biases inherent in strains picked for sequencing and is therefore due to specialization to other environmental variables (i.e. that the clustering actually differentiates rhizosphere vs phyllosphere strains).

### Diverse fungi harbour similar symbionts

Most of the host fungi of EHB strains were isolated from fungi that occurred in healthy leaves on a small number of closely spaced trees in Duke Forest (Durham, NC, USA) [4]. All of the focal EHB strains were isolated as they emerged from fungal cultures. In some cases, we isolated strains that were indistinguishable at the 16S rDNA level, yet occurred in phylogenetically divergent fungi. Whole-genome sequencing could shed light on whether these EHB strains are members of the same clonal group or are just closely related isolates. We also note that it is possible that these strains represent colonization events within the laboratory environment, but the probability of such contamination is very low because careful sterile technique was observed in propagating their host fungi (see Arendt *et al.* [3]).

In the case of *Luteibacter* spp. 9143 and 9145, we found no verified SNPs that could distinguish their genomes from one another (as reflected in branch lengths in Fig. 1b). Although 18 regions differed at a single nucleotide resolution between these two strains, all were within homopolymer tracts and were therefore possibly the product of sequencing errors. These ‘polymorphisms’ alter automatic annotation of the genomes and may account for many of the presumed differences in protein content between the strains. Because these two strains were isolated from highly divergent classes of Ascomycota (Dothideomycetes and Sordariomycetes, respectively; Table 1), we believe that lack of nucleotide diversity is consistent with horizontal transfer of these strains in nature. Such symbiont transfer could have taken place between these two fungal strains, or could represent independent acquisition from a common reservoir (e.g. an environmental source). Although other recent results from our group have demonstrated similar patterns by querying 16S rDNA, phylogenetic signals are strongly reinforced by these whole-genome sequences. More generally, the lack of nucleotide diversity clearly demonstrates that these strains have not been evolving independently in distinct fungal hosts for a long period of time. Notably, we also found a 40 413 bp region that was present within the genome of *Luteibacter* sp. 9143 yet missing from the assembly of *Luteibacter* sp. 9145 (data not shown). This region encoded many phage-associated genes, and therefore likely encodes a prophage. It remains to be seen how the prophage affects the physiology of these strains.

In one additional case, we isolated similar EHB strains from diverse fungal hosts. However, we observed more diversity between *Pantoea* sp. 9140 and *Pantoea* sp. 9133 than between the *Luteibacter* strains mentioned above (as reflected in branch lengths in Fig. 1e): we found 21 SNPs across conserved regions and alignable regions. Moreover, 10 of these SNPs appeared to be true nucleotide polymorphisms because they were not associated with repetitive nucleotide tracts. *Pantoea* sp. 9140 contained additional sequences (11 970 bp on one contig and 171 396 bp on a separate contig) that did not appear to be present in the genome of *Pantoea* sp. 9133. Taken together, comparison of EHB *Luteibacter* and *Pantoea* strains demonstrates that closely related bacteria can be found across divergent fungi, consistent with the lack of strict-sense cocladogenesis observed with natural hosts [3, 4, 13].

### Genomes of these EHB are not reduced

The genome sizes for many intracellular bacteria, including most known EHB, are drastically smaller than those of closely related free-living species (e.g. [14, 42]). Reductions in genome size are thought to be a product of reduced selection pressures on deleterious mutations due to repeated population bottlenecks, a deletion bias for bacterial genomes and lack of selection to maintain physiological pathways made redundant because they are encoded by the host [21]. It is also possible that genomes may be directly streamlined by natural selection as a way to optimize metabolic efficiency [43]. As such, a reduction in genome size compared to closely related bacteria speaks to ecological and evolutionary pressures experienced by intracellular bacteria and therefore provides evidence of selection pressures due to particular lifestyles.

**Fig. 4.**
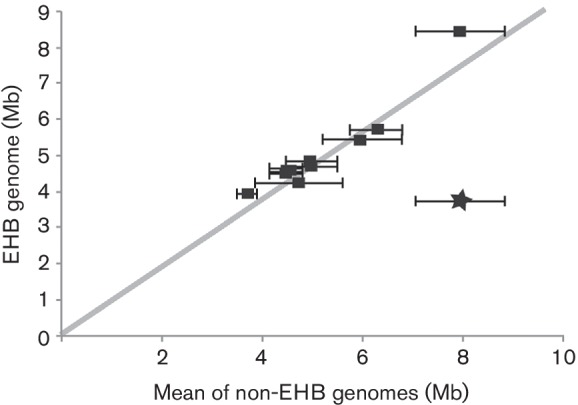
Absence of genome reduction in facultative endohyphal bacteria. Whole-genome sizes for each of the focal EHB strains are plotted on the *y*-axis; mean genome sizes for a diverse suite of related bacteria (all other non-EHB bacteria listed in Figs 1, 2 and 3) are plotted on the *x*-axis. Error bars indicate 1 sd for the ‘non-symbiont’ bacteria against which each ‘symbiont’ genome was plotted. EHB bacteria described in this report are plotted as black squares, while a previously described EHB (*B. rhizoxinica*) is plotted as a star.

We compared the genome size of 11 EHB to closely related, non-EHB strains to test for reduction of genome size (Fig. 4). We also included the genome of *B. rhizoxinica* and used the same comparisons to demonstrate the signal for a known instance of genome reduction. In all but one case, genome sizes for our focal EHB fell essentially within the range of genome sizes for related, free-living bacteria. If anything, the genomes for EHB bacteria may have been larger than expected based on those of their relatives. We therefore saw little evidence that these EHB have generally experienced widespread genome reduction.

### Absence of conserved systems known to direct intimate interdomain interactions

In established systems of bacterial–fungal symbiosis, intimate interactions are usually carried out through the action of various bacterial secretion systems [16]. Indirect interactions are carried out in Gram-negative and Gram-positive bacteria by type I, II and V secretion systems, which secrete substrates outside of cells [44]. Increasingly intimate interactions are largely carried out in Gram-negative bacteria through the actions of type III, IV and VI secretion systems, which translocate substrates (effector proteins) directly into recipient cells [44]. Both type II (for the secretion of chitinase) and type III secretion systems have been implicated in the establishment and maintenance of the *Burkholderia–Rhizopus* interaction [14–16]. Likewise, type III, IV and VI secretion systems are important in interactions between bacteria and single-celled eukaryotes, such as amoebae [45–47].

We queried all 11 complete genomes and those of non-EHB strains for evidence of secretion systems possibly involved in establishment of fungal symbiosis using the JGI’s online annotation tools (Figs 1, 2 and 3). General secretion pathways (types I and II) are likely found within all of these genomes, as expected based on their general presence across a majority of Gram-negative bacteria isolated in culture [44, 48, 49]. Almost all strains except *Curtobacterium* and *Rhizobium* appeared to encode basic type I systems. All 11 bacteria appeared to encode both the Sec and Tat translocation systems, whereas only a subset of these had the genetic potential to create outer-membrane proteins associated with type II secretion (Figs 1, 2 and 3).

A more complex pattern was observed in regard to ‘translocation’-based systems. Genomes of only two EHB examined here (*Erwinia* sp. 9145 and *Burkholderia* sp. 9120) appeared to encode type III secretion systems, with the *Burkholderia* genome likely encoding two separate systems (Figs 1 and 2). In each case, these systems were found also in closely related non-EHB strains. Type IV systems were encoded by many of these genomes, with *Luteibacter* sp. 9133 and *Rhizobium* sp. 9140 appearing to encode two separate systems that also could be found in closely related non-EHB strains (Figs 1 and 3). Because the *Luteibacter* genomes each assembled into one contig, it is likely that there were no plasmids present within these strains and therefore that the type IV secretion systems were encoded by the chromosome. We also note that a type IV system in *Xanthomonas*, closely related to *Luteibacter*, can be utilized to kill other bacterial strains [50]. In contrast, the genome sequence for the *Rhizobium* strain is split into seven distinct contigs, which is expected because related strains contain multiple secondary replicons [51]. However, in *Rhizobium* sp. 9140 both type IV systems were present on smaller plasmids, which suggests that they encode a plasmid transfer system.

The type VI systems encoded by these genomes were also difficult to characterize. On one hand, all focal *Luteibacter* strains and the *Erwinia* sp. 9145 strain appeared to encode one type VI system each, whereas both *Pantoea* strains appeared to encode two distinct systems on the main chromosome (Fig. 1). The *Burkholderia* strain appeared to encode four separate systems, as well as 12 different VgrG proteins and 4 Hcp proteins, higher in number than in closely related non-EHB (Fig. 2). This pattern is particularly intriguing because VgrG and Hcp protein families form the basis of and can be secreted by type VI systems to modify target cells [52]. The greater diversity of these protein families therefore suggests that EHB strains can kill or modify a wider array of target cells. The relatively high diversity of these type VI systems and predicted effectors suggests that *Burkholderia* sp. 9120 interacts with a greater number of other microbes, either through cooperation or competition, than closely related strains. Three of these systems were encoded by the main chromosome, whereas one appeared to be on a smaller contig (likely a plasmid or mini-chromosome). Interestingly, another independently evolved, fungus-associated strain, *B. terrae,* also appeared to encode a higher number of type VI secretion systems than the free-living relatives analysed in this report.

### Ecological inferences from whole-genome sequences of diverse EHB

The preponderance of genomic and phylogenetic data within this report provides the basis for several emergent hypotheses regarding the lifestyles of facultative bacteria found inside the hyphae of Ascomycota that occur as foliar endophytes. First, the genomes of EHB described in this report differed markedly than those of previously characterized obligate EHB in terms of genome size and some genomic features associated with host interaction. This was true both for distantly related strains and for closely related taxa such as *B. rhizoxinica* and *Burkholderia* sp. 9120 (Fig. 2). The genome of *B. rhizoxinica* was nearly half the size of that of *Burkholderia* sp. 9120 and was dramatically smaller than that of most other *Burkholderia* sequenced to data (Fig. 2; [53]). Additionally, the genome of *Burkholderia* sp. 9120 lacked annotated chitinase genes that are considered critical for *B. rhizoxinica* to establish symbiotic associations with its fungal host. It is also noteworthy that *Burkholderia* sp. 9120 maintained four different type VI secretion systems and one type IV secretion system, while both types of systems were absent *B. rhizoxinica*.

The data reported here also demonstrate that facultative EHB bacteria are, in all cases but *Burkholderia* sp. 9120, typically close relatives of strains associated with and likely found in association with plants (Figs 1, 2 and 3). Both EHB *Pantoea* strains (*Pantoea* sp. 9133 and *Pantoea* sp. 9140) were nearly identical to *Pantoea vagans*, *Erwinia* sp. 9145 is a close relative of *Erwinia oleae*, and all other sequenced strains of *Luteibacter* have come from plant-associated samples (Fig. 1). *Rhizobium* sp. 9140 is a member of a clade whose other members are leaf/root-associated strains (Fig. 3). The closest sequenced relatives of EHB *Curtobacterium* sp. 9128 and *Massilia* sp. 9096 were isolated from the rhizosphere of poplar and leaves of *Arabidopsis,* respectively (Figs 2 and 3). While these comparisons may ultimately be biased by which strains have been chosen for sequencing, and some strains isolated from rhizosphere or phyllosphere samples may themselves actually be EHB, plant-associated bacteria and EHB strains often share common ancestry.

The absence of genome reduction within the EHB considered here (Fig. 4) is consistent with laboratory studies suggesting that facultative EHB are gained and lost readily from fungi, that fungi are capable of major metabolic activity in the absence of the bacteria, that the bacteria can be isolated on standard laboratory media, and that they are transmitted horizontally (see [3, 4, 11]). It is therefore plausible that these strains do not experience drastic population bottlenecks during transmission, and that diverse genomic architecture needed for survival outside of hosts has been maintained.

Overall the data suggest two inferences regarding the lifestyle of EHB. First, it is possible that subsets of the strains described in this report have adapted to include the endohyphal niche as part of their lifestyle. Alternatively, they may be members of the rhizosphere/phyllosphere that have incidentally found their way into the hyphae of Ascomycota. Indeed, the ease at which such interactions occur could explain why bacteria such as *Ralstonia* species can invade cells of and interact with a broad diversity of soil-borne fungi even though there is no known history of association [20]. Furthermore, although patterns of horizontal transfer have been suggested by previous data sets querying 16S rDNA sequences [13], comparison of whole-genome sequences definitively demonstrates that nearly identical strains can be found within distinct fungal hosts. From these associations, it is apparent that diverse bacterial strains can either readily find their way into the hyphae of a diverse set of fungal endophytes or that these strains are readily horizontally transferred across these hosts. Indeed, we have shown that closely related *Luteibacter* strains can colonize a variety of these diverse fungal endophytes given the opportunity ([12]; D. A. Baltrus, unpublished).

### Conclusions

Herein, we report nearly complete genome sequences for a diverse suite of bacteria found living inside the hyphae of endophytic fungi representing the diverse fungal phylum, Ascomycota. Phylogenetic analyses suggest that these EHB are distinct from previously described EHB, and that the endofungal lifestyle has convergently and independently evolved over short time scales both across diverse bacterial lineages, and in closely related taxa such as *Pantoea* and *Erwinia*. We evaluated these genomes for the presence/absence of sequences relevant to encoding structures involved in interdomain interactions between bacteria and eukaryotes. Although each strain contained structures that could mediate interactions with fungi, no general mechanism was conserved across strains.

More broadly, these genome sequences provide insights into the ecology of these facultative EHB in that no genome reduction is apparent and different classes of fungi can harbour very similar bacteria. Both pieces of evidence suggest that horizontal transmission is the dominant mode of acquisition by fungal hosts in nature.

Fungal endophytes, and the Ascomycota that they represent, are largely thought to be hyperdiverse [10]. Our results suggest that diverse bacteria have independently evolved the mechanisms needed to infect these ecologically and economically important fungi. Shaffer *et al*. [13] also recently showed that diverse bacteria can be found within fungal endophytes associated with seeds and leaves, but that not all isolates of a given fungal genotype may be colonized in natural conditions. In this way, fungi may represent a special case wherein axenic and colonized versions of the same eukaryotic host may exist in nature outside of sterile chambers. Given the capacity of EHB to influence fungal phenotypes [6, 8, 12], these findings illuminate a new dimension of fungal biodiversity.

## Data Bibliography

1. Baltrus DA, Dougherty K, Arendt KR, Shapiro N, Kyrpides N *et al*. Genome Assembly Summary Files, Figshare, https://dx.doi.org/10.6084/m9.figshare.4123320 (2016).
2. Baltrus DA, Dougherty K, Arendt KR, Shapiro N, Kyrpides N *et al*. Strain Accession Information, KEGG Pathway Classification and Genome Size Calculations, Figshare, https://dx.doi.org/10.6084/m9.figshare.4123320 (2016).
3. Baltrus DA, Dougherty K, Arendt KR, Shapiro N, Kyrpides N *et al*. Protein Alignments and Output from Phylogenetic Comparisons, Figshare, https://dx.doi.org/10.6084/m9.figshare.4123320 (2016).
4. Baltrus DA, Dougherty K, Arendt KR, Shapiro N, Kyrpides N *et al*. Genome Assembly for Burkholderia sp. 9120. GenBank accession no. JQNA00000000.1, www.ncbi.nlm.nih.gov/assembly/GCF_000745015.1/ (2015).
5. Baltrus DA, Dougherty K, Arendt KR, Shapiro N, Kyrpides N *et al*. Genome Assembly for Curtobacterium sp. 9128. GenBank accessionno. LT576451.1. https://www.ncbi.nlm.nih.gov/nuccore/LT576451.1/ (2016).
6. Baltrus DA, Dougherty K, Arendt KR, Shapiro N, Kyrpides N *et al*. Genome Assembly for *Erwinia* sp. 9145. GenBank accession no. JQNE00000000.1, www.ncbi.nlm.nih.gov/assembly/GCF_000745075.1/ (2015).
7. Baltrus DA, Dougherty K, Arendt KR, Shapiro N, Kyrpides N *et al*. Genome Assembly for *Luteibacter* sp. 9133. GenBank accession no. JUHO00000000.1, www.ncbi.nlm.nih.gov/assembly/GCF_000800155.1/ (2015).
8. Baltrus DA, Dougherty K, Arendt KR, Shapiro N, Kyrpides N *et al*. Genome Assembly for *Luteibacter* sp. 9135. GenBank accession no. JQNB00000000.1, www.ncbi.nlm.nih.gov/assembly/GCF_000745005.1/ (2015).
9. Baltrus DA, Dougherty K, Arendt KR, Shapiro N, Kyrpides N *et al*. Genome Assembly for *Luteibacter* sp. 9143. GenBank accession no. JQNL00000000.1, www.ncbi.nlm.nih.gov/assembly/GCF_000745235.1/ (2015).
10. Baltrus DA, Dougherty K, Arendt KR, Shapiro N, Kyrpides N *et al*. Genome Assembly for *Luteibacter* sp. 9145. GenBank accession no. JQND00000000.1, www.ncbi.nlm.nih.gov/assembly/GCF_000745055.1/ (2015).
11. Baltrus DA, Dougherty K, Arendt KR, Shapiro N, Kyrpides N *et al*. Genome Assembly for *Massilia* sp. 9096. GenBank accession no. JQNN00000000.1, www.ncbi.nlm.nih.gov/assembly/GCF_000745265.1/ (2015).
12. Baltrus DA, Dougherty K, Arendt KR, Shapiro N, Kyrpides N *et al*. Genome Assembly for *Pantoea* sp. 9133. GenBank accession no. FKKH00000000.1, www.ncbi.nlm.nih.gov/assembly/ GCA_900067205.1/ (2016).
13. Baltrus DA, Dougherty K, Arendt KR, Shapiro N, Kyrpides N *et al*. Genome Assembly for *Pantoea* sp. 9140. GenBank accession no. JQNO00000000.1, www.ncbi.nlm.nih.gov/assembly/GCF_000745295.1/ (2015).
14. Baltrus DA, Dougherty K, Arendt KR, Shapiro N, Kyrpides N *et al*. Genome Assembly for *Rhizobium* sp. 9140. GenBank accession no. FJUR00000000.1, www.ncbi.nlm.nih.gov/assembly/ GCA_900067135.1/ (2016).
